# The differential effects of eicosapentaenoic acid and docosahexaenoic acid on cardiovascular risk factors: an updated systematic review of randomized controlled trials

**DOI:** 10.3389/fnut.2024.1423228

**Published:** 2024-09-30

**Authors:** Gyu Yeong Choi, Philip C. Calder

**Affiliations:** ^1^Faculty of Medicine, School of Human Development and Health, University of Southampton, Southampton, United Kingdom; ^2^NIHR Southampton Biomedical Research Centre, University Hospital Southampton NHS Foundation Trust and University of Southampton, Southampton, United Kingdom

**Keywords:** blood lipids, triglycerides, cholesterol, blood pressure, inflammation

## Abstract

Cardiovascular disease remains a major global health concern. The combination of the omega-3 fatty acids eicosapentaenoic acid (EPA) and docosahexaenoic acid (DHA) has been shown to beneficially modify a range of cardiovascular risk factors. However, whether EPA and DHA have differential effects or potencies is currently unclear. A systematic review of randomized controlled trials (RCTs) that compared ≥2 g/day of near pure EPA and DHA was conducted. A total of 24 publications from nine unique RCTs were included. EPA and DHA both lower triglyceride levels, with DHA most likely having a slightly greater effect. Furthermore, both EPA and DHA increase high density lipoprotein (HDL) 2 cholesterol, which is cardioprotective, with the increase being greater with DHA. DHA appears to increase low density lipoprotein (LDL) cholesterol; however, DHA also increases LDL particle size, which would render LDL less atherogenic. DHA seems more effective than EPA in decreasing heart rate and blood pressure. Both EPA and DHA alter platelet function decreasing thrombogenicity, although they may have different actions on platelets. Both EPA and DHA decrease F2-isoprostanes, interpreted as a reduction in oxidative stress. They both decrease inflammatory gene expression and promote an anti-inflammatory oxylipin profile. These are all favorable effects with regard to cardiovascular disease risk. Effects of EPA and DHA on blood glucose are inconsistent. This review is constrained by the small number of high quality RCTs that directly compare EPA to DHA and report on outcomes other than blood lipids. There is a need for additional high-quality research to assess the independent effects of EPA and DHA on cardiovascular risk factors (e.g., inflammation, blood pressure, vascular function, platelet function) in larger and more diverse study populations.

## Introduction

Cardiovascular disease (CVD) remains the leading cause of mortality worldwide, with around 18.56 million deaths globally in 2019 (1). Of particular concern are the modifiable risk factors that contribute to CVD, which include (but are not limited to) elevated blood cholesterol and triglycerides, inflammation, hypertension, and diabetes. The omega-3 (n-3) polyunsaturated fatty acids eicosapentaenoic acid (EPA) and docosahexaenoic acid (DHA) have emerged as potential interventions to control these risk factors (2–4) resulting in cardioprotective properties (3–5). Although fish is the main dietary source of EPA and DHA, the study of these fatty acids has been helped by the ready availability of supplemental forms and their effects have most often been studied as the combination of EPA and DHA. This combination has been shown to lower triglycerides (6), blood pressure (7), inflammation (8–10) and heart rate (11, 12), to raise high-density lipoprotein (HDL) cholesterol (2), to improve vascular reactivity (13–15), and to reduce platelet reactivity and thrombosis (16). They may also raise low density lipoprotein (LDL) cholesterol (2) and fasting blood glucose (17). However, whether EPA and DHA have differential effects or potencies on risk factors for CVD is uncertain, in part because most trials have focussed on the effects of EPA and DHA when used in combination. It is of interest to know which is the more effective, EPA or DHA, and this information would be useful for regulators, industry and consumers. A small number of trials have been performed that directly compare the effects of pure EPA with pure DHA; these trials were subject to a systematic review published in 2018 (18) that focused on the differential effects of EPA and DHA using the findings from six randomized controlled trials (RCTs) reported in 18 publications (19–36). Knowing that there have been several more trials and publications on this topic published since then, this new systematic review aims to update the previous one and provide a comprehensive and up-to-date analysis of the current literature that specifically compares the effects of EPA and DHA on risk factors for CVD. While there is not a universally agreed-upon recommended dosage for EPA and DHA, this systematic review employed a strict inclusion criterion of ≥2 g per day due to evidence suggesting that this threshold shows favorable impacts on several relevant risk factors (37–39). Furthermore, trials included in this review needed to have used ≥90% of n-3 polyunsaturated fatty acids as either EPA or DHA in order to avoid, as best as possible, the biological effects of the “other” n-3 polyunsaturated fatty acid. These inclusion criteria are consistent with those of the previous systematic review (18).

## Materials and methods

### Literature search

This systematic review was conducted according to the principles of Preferred Reporting for Systematic Reviews and Meta-Analyses (PRISMA) (40). Searches were conducted in October 2023 in PubMed (2017 to October 2023), EMBASE (2017 to October 2023) and CINAHL (2017 to October 2023) databases. Earlier years were not searched because eligible publications up until 2017 had been identified in the previous systematic review (18). Search terms used included: “EPA,” “DHA,” “eicosapentaenoic acid,” “docosahexaenoic acid” together with “blood lipid,” “lipid,” “triglyceride,” “cholesterol,” “LDL,” “HDL,” “lipoprotein,” “blood pressure,” “inflamm*,” “interleukin-6,” “IL-6,” “C-reactive protein,” “CRP,” “vascular,” “heart rate,” “cardiovascular,” “cardiometabolic.” These search terms are consistent with those used in the previous systematic review (18). The full search strategies for the three databases are shown in Supplementary Table 1. This systematic review was not registered because it was conducted for educational purposes.

### Publication selection

**Figure 1:**
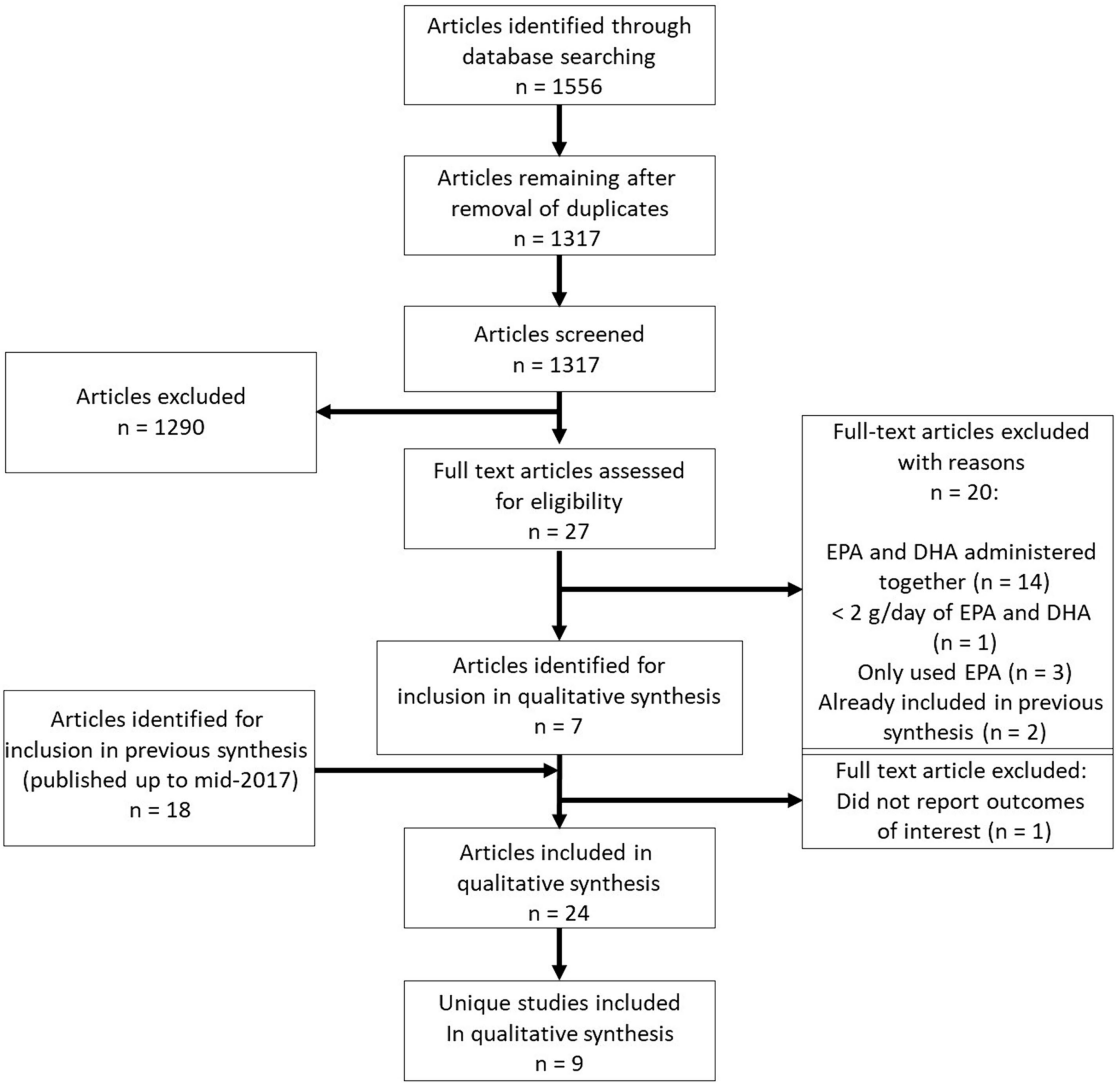
PRISMA flow chart showing selection of publications for inclusion in the systematic review.

Publications had to meet the following criteria to be included in the qualitative synthesis: study must be in humans; study must compare pure or near pure EPA and DHA; study design must be a RCT; dose of EPA and DHA used must be ≥2 g per day; study has to include outcomes of interest (predefined risk factors for CVD); study must be published in the English language; study has to be available as full text. Publications from a previous systematic review (18) on the comparative effects of EPA and DHA on risk factors for CVD that included literature published up to 2017 were also included for qualitative synthesis as shown on the PRISMA flow chart (Figure 1). Publications which met the following criteria were excluded: animal studies; *in vitro* studies; dose of EPA and DHA used did not meet ≥2 g per day; outcomes of interest not stated or included; study was not an RCT; not published in the English language; study compared other interventions alongside EPA and DHA.

### Data extraction

The data extracted for each trial included the study design, study population, sample size, dosage levels of EPA, DHA and placebo, study duration and relevant outcomes measured.

### Quality assessment

The “parent” publication for each trial was assessed for methodological quality and validity using the Jadad scale (41). Risk of bias was assessed for each individual publication using the Cochrane Risk of Bias tool for RCTs (42).

## Results

### Identification of included publications

From the electronic literature searches, 1,566 publications of potential relevance were identified. Of these, 249 were removed due to being duplicates and 1,317 were assessed for eligibility based on title and abstract. From these, 1,290 were excluded due to not meeting the eligibility criteria. Based on the abstracts of the remaining 27 publications, 20 were excluded due to the following reasons: trials described in 14 publications administered EPA and DHA together, 1 trial utilized <2 g/day of EPA and DHA, trials reported in three publications administered EPA only, and two publications had already been recorded in the previous systematic review (35, 36). One of these latter publications included in the previous review (35) did not report outcomes of relevance and so was excluded. Hence, seven newly-identified publications were included for qualitative synthesis (43–49). Two of these newly-identified publications (43, 44) report additional results from a trial with publications included in the previous review. Five of the newly-identified publications (45–49) report results from three trials not included in the previous review. From the previous systematic review, 17 publications were also included (19–34, 36). Therefore, a total of 24 publications were included in this systematic review (Figure 1). A number of these publications were from the same trials; hence a total of nine unique RCTs were identified for inclusion; results from six unique RCTs were included in the previous review.

### Characteristics of included studies

Table 1 summarizes the characteristics of the nine unique trials including design, population, sample size, dose of EPA, DHA and placebo used, duration, outcomes reported and Jadad score (based on the “parent” publication).

**Table 1:** Summary of characteristics of the nine included studies.

Study	Country	Study design and population	Sample size	Dose of EPA/DHA/Placebo (g/day)	Duration (Weeks)	Outcomes	Jaded score based on the “parent” publication
Grimsgaard et al. (19, 20)	Norway	Double-blind, randomized controlled trial. Healthy men.	*n* = 75 (EPA) *n* = 72 (DHA) *n* = 77 (corn oil) *n* = 224 (total)	3.8 (EPA) 3.6 (DHA) 4.0 (corn oil)	7	Serum lipids (total cholesterol, LDL cholesterol, HDL cholesterol, ApoA1, ApoB, triglycerides) Haemodynamics (blood pressure, heart rate, left ventricular function)	5 (19)
Mori et al. (21–24), Mas et al. (25)	Australia	Double-blind, randomized controlled trial. Overweight mildly hyperlipidaemic men.	*n* = 19 (EPA) *n* = 17 (DHA) *n* = 20 (olive oil) *n* = 56 (total)	3.8 (EPA) 3.7 (DHA) 3.0 (olive oil)	6	Serum lipids (total cholesterol, LDL cholesterol, HDL cholesterol, triglycerides, LDL particle size) Oxidative stress markers (urinary and plasma F_2_-isoprostanes) Glycaemic control (serum glucose, serum insulin) Haemodynamics (blood pressure, heart rate, vascular reactivity)	3 (21)
Nestel et al. (30)	Australia	Double-blind, randomized controlled trial. Dyslipidaemic subjects.	*n* = 12 (EPA) *n* = 12 (DHA) *n* = 14 (olive oil) *n* = 38 (total)	3.0 (EPA) 2.8 (DHA) 2.8 (olive oil)	7	Plasma lipids (total cholesterol, LDL cholesterol, HDL cholesterol, VLDL triglycerides, total triglycerides) Haemodynamics (blood pressure, pulse pressure, heart rate, total vascular resistance, systemic arterial compliance)	4 (30)
Woodman et al. (26–28), Mori et al. (29), Mas et al. (25)	Australia	Double-blind, randomized controlled trial. People with type-2 diabetes and treated for hypertension.	*n* = 17 (EPA) *n* = 18 (DHA) *n* = 16 (olive oil) *n* = 51 (total)	3.8 (EPA) 3.7 (DHA) 3.0 (olive oil)	6	Serum lipids (total cholesterol, LDL cholesterol, HDL cholesterol, triglycerides, LDL particle size) Haemodynamics (blood pressure) Glycaemic control (serum glucose, insulin, C-peptide, insulin sensitivity, insulin secretion) Plasma inflammatory markers (CRP, TNF-*α*, IL-6) Platelet, fibrinolytic and vascular function (collagen and PAF-stimulated platelet aggregation, collagen-stimulated thromboxane release, plasma tPA and PAI-1 antigen, von Willebrand factor, P-selectin, brachial artery dilatation) Oxidative stress markers (urinary and plasma F_2_-isoprostanes)	3 (26)
Park and Harris (31, 32), Park et al. (33)	USA	Double-blind, randomized controlled trial. Healthy participants.	*n* = 11 (EPA) *n* = 11 (DHA) *n* = 11 (safflower oil) *n* = 33 (total)	3.8 (EPA) 3.8 (DHA) 4.0 (safflower oil)	4	Plasma lipids (total cholesterol, HDL cholesterol, LDL cholesterol, VLDL cholesterol, triglycerides, chylomicron size, ApoB48, ApoB100, chylomicron clearance) Plasma LPL and hepatic lipase activities Platelet count and platelet volume	3 (31)
Allaire et al. (34, 43, 44), Vors et al. (36)	Canada	Double-blind randomized controlled crossover study with 9-week washout. Healthy subjects with abdominal obesity and subclinical inflammation.	*n* = 121 (EPA) *n* = 123 (DHA) *n* = 125 (corn oil) *n* = 125 (total)	2.7 (EPA) 2.7 (DHA) 3.0 (corn oil)	10	Plasma lipids (total cholesterol, LDLcholesterol, HDL cholesterol, ApoB, triglycerides) Plasma triglyceride-lowering responders LDL particle size, % small-dense LDL PCSK9 *In vivo* kinetics of ApoB100-containing lipoproteins (in a subset of 19) Whole blood expression of lipid metabolism genes (HMGCoA reductase, LDLR, SREBP1c, SREBP2) (in a subset of 44) Plasma inflammatory markers (CRP, IL-6, IL-18, TNF-α, adiponectin) Whole blood expression of inflammatory genes (PPARA, TNFA, CD14, TRAF3, CCL2, IL10, IL1B, IL1RN, NFKB, TNFRSF1A) (in a subset of 44)	5 (34)
Klingel et al. (45), Lee et al. (46)	Canada	Double-blind, randomized controlled trial. Healthy men and women.	*n* = 29 (EPA) *n* = 30 (DHA) *n* = 30 (olive oil)	3.0 (EPA) 3.0 (DHA) 3.0 (olive oil)	12	Serum lipids (total cholesterol, HDL cholesterol, triglycerides) Serum glucose Serum LPL activity Marker of *de novo* lipogenesis Haemodynamics (blood pressure, heart rate, cardiac function)	5 (46)
So et al. (47, 48)	USA	Double-blind randomized controlled crossover study with 10-week washout. Men and postmenopausal women (age 50–75 years) with chronic inflammation.	*n* = 21 (EPA) *n* = 21 (DHA) *n* = 21 (high-oleic sunflower oil)	3.0 (EPA) 3.0 (DHA) 3.0 (high-oleic sunflower oil)	10	Plasma lipids (total cholesterol, LDL cholesterol, HDL cholesterol, ApoB, ApoA1, triglycerides) Plasma activities of LPL, CETP and LCAT Plasma inflammatory markers (CRP, TNF-α, IL-6, MCP-1, IL-10) Cytokine gene expression by LPS-stimulated blood monocytes (TNFA, IL6, MCP1, IL10) Plasma oxylipin profile	4 (47)
Pisaniello et al. (49)	Australia	Double-blind randomized controlled trial. Healthy participants.	*n* = 10 (EPA) *n* = 10 (DHA) *n* = 10 (fish oil concentrate) *n* = 10 (palm oil, sunflower oil, rapeseed oil, and fish oil)	4.0 (EPA) 4.0 (DHA) 4.0 (fish oil or mixed oils)	4	Serum lipids (total cholesterol, LDL cholesterol, HDL cholesterol, triglycerides) Serum inflammatory marker (CRP) Effect of serum on TNF-stimulated inflammatory gene expression in endothelial cells (ICAM, VCAM, CCL2, NFKB subunit) Haemodynamics (blood pressure, heart rate)	5 (49)

Apo, apolipoprotein; CCL, chemokine (C-C motif) ligand; CETP, cholesteryl ester transfer protein; CRP, C-reactive protein; HDL, high density lipoprotein; HMGCoA, hydroxymethylglutaryl coenzyme A; ICAM, intercellular adhesion molecule; IL, interleukin; LCAT, lecithin cholesterol acyl transferase; LDL, low density lipoprotein; LDLR, low density lipoprotein receptor; LPL, lipoprotein lipase; LPS, lipopolysaccharide; MCP, monocyte chemoattractant protein; NFKB, nuclear factor kappa-light-chain-enhancer of activated B cells; PAF, platelet activating factor; PAI, plasminogen activator inhibitor; PCSK9, proprotein convertase subtilisin/kexin type 9; PPAR, peroxisome proliferator activated receptor; SREBP, sterol receptor element binding protein; TNF, tumor necrosis factor; TNFRSF, tumor necrosis family receptor superfamily; tPA, tissue plasminogen activator; TRAF, tumor necrosis factor receptor associated factor; VCAM, vascular cell adhesion molecule; VLDL, very low density lipoprotein.

The sample size of the included trials varied between 21 and 224, with a total of 763 participants studied overall. Four trials included healthy participants and one, participants described as healthy with abdominal obesity and chronic inflammation. Other trials included those with overweight and hyperlipidaemia, with dyslipidaemia, with chronic inflammation or with diabetes and being treated for hypertension. The dosage of EPA and DHA given ranged from 2.8 to 4 g/day. The trial durations varied between 4 and 12 weeks (mean 7 weeks). All trials provided EPA, DHA, and placebo in capsules. Placebos used included olive oil (*n* = 4 trials), corn oil (*n* = 2), high oleic sunflower oil (*n* = 1), mixed oils (*n* = 1) and safflower oil (*n* = 1). Trial locations varied and included Australia (*n* = 4 trials), Canada (*n* = 2), USA (*n* = 2) and Norway (*n* = 1). Jadad scores, based on the “parent” publication, varied from 3 to 5, with 4 studies receiving the maximum score of 5. Reasons for not achieving the maximum score were lack of information on method of randomization and/or on method of blinding.

A summary of risk of bias, according to the Cochrane criteria for RCTs, is shown in Table 2. This analysis was completed for each individual publication because the details of different publications from the same trial (e.g., participant numbers) sometimes varied. Most publications, including all publications from 6 of the trials, had a low risk of bias. Reasons for concern are listed, where relevant, in Table 2.

**Table 2:** Risk of bias assessment for each individual publication.

Study	Bias arising from the randomization process	Bias due to deviations from intended intervention	Bias due to missing outcome data	Bias in measurement of the outcome	Bias in selection of the reported result	Overall bias	Comments
Grimsgaard et al. (19)	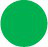	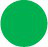	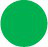	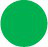	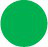	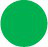	
Grimsgaard et al. (20)	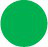	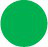	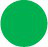	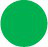	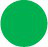	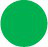	
Mori et al. (21)	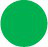	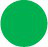	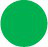	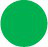	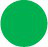	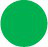	
Mori et al. (22)	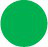	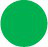	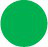	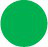	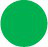	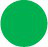	40 participants from the 59 randomized and 56 completers agreed to the experimental procedure
Mori et al. (23)	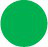	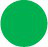	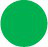	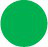	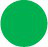	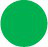	
Mori et al. (24)	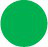	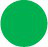	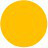	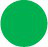	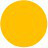	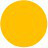	Baseline values for the outcome are not provided. It is not stated how many participants’ data are reported (i.e., sample size is not specified). It is not stated whether this outcome was pre-specified.
Mas et al. (25)	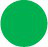	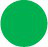	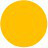	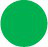	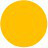	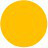	It is not stated how many participants’ data are reported (i.e., sample size is not specified). It is not stated whether this outcome was pre-specified.
Woodman et al. (26)	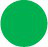	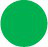	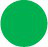	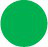	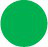	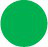	
Woodman et al. (27)	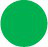	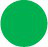	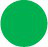	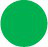	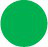	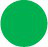	
Woodman et al. (28)	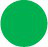	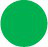	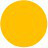	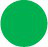	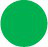	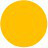	It is not stated how many participants’ data are reported (i.e., sample size is not specified).
Mori et al. (29)	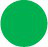	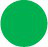	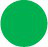	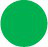	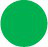	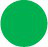	
Nestel et al. (30)	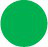	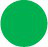	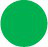	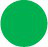	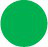	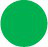	
Park and Harris (31)	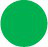	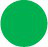	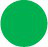	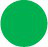	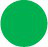	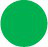	
Park and Harris (32)	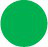	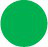	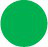	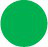	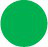	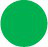	
Park et al. (33)	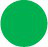	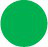	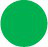	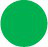	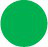	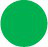	
Allaire et al. (34)	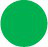	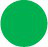	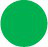	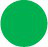	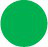	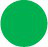	
Allaire et al. (43)	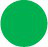	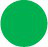	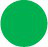	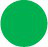	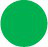	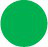	Completed in a subset of the first 20 participants
Allaire et al. (44)	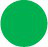	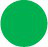	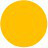	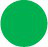	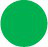	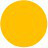	It is not stated how the sub-set of 44 participants for gene expression analysis was selected
Vors et al. (36)	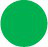	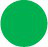	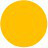	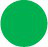	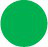	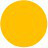	It is not stated how the sub-set of 44 participants was selected
Lee et al. (46)	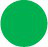	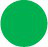	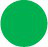	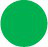	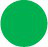	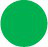	
Klingel et al. (45)	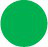	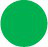	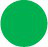	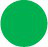	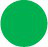	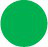	
So et al. (47)	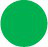	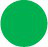	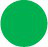	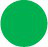	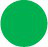	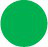	
So et al. (48)	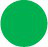	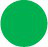	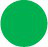	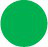	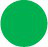	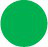	
Pisanello et al. (49)	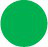	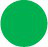	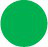	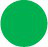	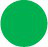	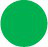	

Green circle = low risk of bias; Amber circle = some concerns.

### Effects of EPA and DHA on the concentration of EPA and DHA in blood pools

Table 3 summarizes the publications that report on the EPA and DHA concentration in different blood pools. Most publications report fatty acids as % of total fatty acids in the pool, although one reports absolute concentration (19). Of the 12 publications listed, six report fatty acids in plasma or plasma or serum phospholipids (19, 21, 26, 30, 34, 48), while three report fatty acids in platelets (23, 29, 31), two - both from the same trial - in erythrocytes (45, 46) and one in whole blood (49). Supplementing with near pure EPA increases the EPA content of all pools reported on, while supplementing with near pure DHA increases the DHA content of all pools reported on (Table 3). Furthermore, most studies report that supplementing near-pure DHA increases the EPA content of the pools reported on. However, effects of near-pure EPA on DHA concentration are inconsistently observed, with some studies observing a decrease in DHA (19, 23, 26, 29), others little or no change (21, 30, 31, 34, 45, 46, 48, 49). No studies report a significant increase in DHA when EPA is supplemented.

**Table 3:** Summary of the effects of EPA and DHA supplementation on blood and cell levels of these fatty acids.

Study	Study population	Dose of EPA or DHA (g/day)	Duration (Weeks)	Where fatty acids measured	Units	Group	EPA before supplementation	EPA after supplementation	DHA before supplementation	DHA after supplementation
Grimsgaard et al. (19)	Healthy men	3.8 (EPA) 3.6 (DHA)	7	Serum phospholipids	μmol/L	EPA	61.4	+182.1	184.0	−28.0
						DHA	59.8	+17.6	185.0	+128.0
Mori et al. (21)	Overweight mildly hyperlipidaemic men	3.8 (EPA) 3.7 (DHA)	6	Plasma phospholipids	%	EPA	1.7	9.8	4.1	4.0
						DHA	1.5	2.3	4.0	10.9
Mori et al. (23)	Overweight mildly hyperlipidaemic men	3.8 (EPA) 3.7 (DHA)	6	Platelets	%	EPA	~0.1	~3.6	~0.2	~ − 0.7
						DHA	~0.1	~0.6	~0.2	~4.3
Nestel et al. (30)	Dyslipidaemic subjects	3.0 (EPA) 2.8 (DHA)	7	Plasma	%	EPA	1.6	9.0	NR	NR but no change
						DHA	1.1	2.7	2.2	7.2
Woodman et al. (26)	People with type-2 diabetes and treated for hypertension	3.8 (EPA) 3.7 (DHA)	6	Plasma phospholipids	%	EPA	1.6	~10.2 (+540%)	4.3	~4.0 (−7%)
						DHA	1.7	~2.8 (+64%)	4.3	~11.0 (+156%)
Mori et al. (29)	People with type-2 diabetes and treated for hypertension	3.8 (EPA) 3.7 (DHA)	6	Platelets	%	EPA	1.0	4.8	2.3	1.7
						DHA	1.0	1.5	2.4	5.2
Park and Harris (31)	Healthy participants	3.8 (EPA) 3.8 (DHA)	4	Platelets	%	EPA	0.2	3.3	1.5	1.6
						DHA	0.4	0.4	1.4	4.1
Allaire et al. (34)	Healthy subjects with abdominal obesity and subclinical inflammation	2.7 (EPA) 2.7 (DHA)	10	Plasma phospholipids	%	EPA	NR but 1.1 in control group	6.0	NR but 3.3 in control group	3.3
						DHA	NR but 1.1 in control group	2.1	NR but 3.3 in control group	8.1
Klingel et al. (45), Lee et al. (46)	Healthy men and women	3.0 (EPA) 3.0 (DHA)	12	Erythrocytes	%	EPA	0.5	3.9	3.0	2.5
						DHA	0.5	1.2	2.9	7.2
So et al. (48)	Men and postmenopausal women (age 50–75 years) with chronic inflammation	3.0 (EPA) 3.0 (DHA)	10	Plasma phospholipids	%	EPA	0.7	5.3	2.8	3.1
						DHA	0.7	1.6	2.8	7.7
Pisaniello et al. (49)	Healthy participants	4.0 (EPA) 4.0 (DHA)	4	Whole blood	%	EPA	0.8	~2.8 (+253%)	1.9	NR but no change
						DHA	0.5	NR but no change	1.7	~4.2 (+145%)

NR, not reported.

### Comparative effects of EPA and DHA on cardiovascular risk factors

#### Effect of EPA vs. DHA on blood lipids and lipoproteins

All nine trials (13 publications) (19, 23, 26, 28, 30, 32–34, 43–45, 48, 49) included outcomes related to the effect of EPA and DHA on blood lipids (Table 4).

**Table 4:** Summary of findings related to effects of EPA and DHA on blood lipids and lipoproteins.

Study	Population	Control	Effect of EPA vs. control on blood lipids and lipoproteins	Effect of DHA vs. control on blood lipids and lipoproteins	Effect of EPA vs. DHA on blood lipids and lipoproteins
Grimsgaard et al. (19)	Healthy men	Corn oil	↓ Triglycerides (−21%, *p* = 0.0001) ↓ Total cholesterol (−0.15 ± 0.55 mmol/L, *p* < 0.05) ↓ ApoA1 (−0.04 ± 0.10 g/L, *p* < 0.001) ↓ ApoB (−0.03 ± 0.11 g/L, *p* < 0.05) ↑ HDL:ApoA1 (+0.04 ± 0.08, *p* = 0.0001) ↓ Total:HDL cholesterol (−0.13 ± 0.47, *p* = 0.007)	↓ Triglycerides (−26%, *p* = 0.0001) ↑ HDL cholesterol (+0.06 ± 0.13 mmol/L, *p* < 0.001) ↑ HDL:ApoA1 (+0.04 ± 0.07, *p* < 0.001) ↓ Total/HDL cholesterol (−0.19 ± 0.52, *p* < 0.01)	EPA ↓ total cholesterol and ApoA1 more than DHA DHA ↑ HDL cholesterol more than EPA (*p* = 0.009)
Mori et al. (23)	Overweight mildly hyperlipidaemic men	Olive Oil	↓ Triglycerides (−18%, *p* = 0.012) ↓ HDL_3_ cholesterol (−7%, *p* = 0.032) No effect on total, HDL, HDL_2_ or LDL cholesterol No effect on LDL particle size	↓ Triglycerides (−20%, *p* = 0.003) ↑ LDL cholesterol (+8%, *p* = 0.019) ↑ LDL particle size (+0.25 ± 0.08 nm, *p* = 0.002) ↑ HDL_2_ cholesterol (+29%, *p* = 0.004) No effect on total, HDL or HDL_3_ cholesterol	N/A
Woodman et al. (26, 28)	People with type-2 diabetes treated for hypertension	Olive oil	↓ Triglycerides (−19%, *p* = 0.022) ↑ HDL_2_ cholesterol (+16%, *p* = 0.026) ↓ HDL_3_ cholesterol (−11%, *p* = 0.026) No effect on total, LDL- or HDL cholesterol No effect on LDL particle size	↓ Triglycerides (−15%, *p* = 0.022) ↑ HDL_2_ cholesterol (+12%, *p* = 0.05) No effect on total, LDL, HDL or HDL_3_ cholesterol ↑ LDL particle size (+0.26 ± 0.10 nm, *p* = 0.02)	N/A
Nestel et al. (30)	Dyslipidaemic subjects	Olive oil	↓ Total triglycerides (−23%, *p* = 0.026) ↓ VLDL triglycerides (*p* = 0.006) No effect on total, HDL or LDL cholesterol	↓ Total triglycerides (−32%, *p* = 0.026) ↓ VLDL triglycerides (*p* = 0.006) No significant difference in total, HDL or LDL cholesterol	No significant difference between EPA and DHA
Park and Harris (32), Park et al. (33)	Healthy subjects	Safflower oil	No effect on plasma lipids (total, LDL-, HDL- or VLDL cholesterol, triglycerides) ↓ ApoB48 after an oral fat challenge (*p* < 0.001) ↓ ApoB100 after an oral fat challenge (*p* < 0.01) ↓ Chylomicron triglyceride half-life (fed state) (*p* < 0.05) ↓ Chylomicron particle size (−53%, *p* < 0.01) ↑ Pre-heparin LPL activity (47%, *p* < 0.05); no effect on post-heparin LPL activity or hepatic lipase activity ↑ Margination volume in the fasted state (*p* < 0.001)	No effect on plasma lipids (total, LDL-, HDL- or VLDL cholesterol, triglycerides) ↓ ApoB48 after an oral fat challenge (−28%, *p* < 0.001) ↓ ApoB100 after an oral fat challenge (−24%, *p* < 0.01) ↓ Chylomicron triglyceride half-life (fed state) (*p* < 0.05) ↓ Chylomicron particle size (−24%, *p* < 0.01) ↑ Pre-heparin LPL activity (73%, *p* < 0.05); no effect on post-heparin LPL activity or hepatic lipase activity ↑ Margination volume in the fasted state (*p* < 0.001) ↑ Margination volume in the fed state (*p* < 0.05)	No difference between EPA and DHA
Allaire et al. (34, 43, 44)	Healthy subjects with abdominal obesity and subclinical inflammation	Corn Oil	↓ Triglycerides (−12%, *p* < 0.0001) ↑ LDL cholesterol (+2%, *p* = 0.046) ↓ mean LDL particle size ↑ Proportion of small dense LDL ↓ PCSK9 concentrations ↑ VLDL ApoB100 fractional catabolism rate ↓ LDL ApoB100 fractional catabolism rate	↓ Triglycerides (−13%, *p* < 0.0001) ↑ Total cholesterol (+4%, *p* = 0.001) ↑ LDL cholesterol (+7%, *p* < 0.0001) ↑ HDL cholesterol (+8%, *p* < 0.0001) ↓ Cholesterol/HDL cholesterol ratio (−3%, *p* < 0.001) ↑ ApoB (+5%, *p* = 0.02) ↑ mean LDL particle size ↓ Proportion of small dense LDL ↓ PCSK9 concentrations ↑ VLDL ApoB100 fractional catabolism rate	Compared to EPA, DHA ↓ Plasma triglycerides (*p* = 0.005) ↑ Plasma total cholesterol (*p* < 0.001) ↑ Plasma LDL cholesterol (*p* = 0.04), more so in men than women (*p* = 0.046) ↑ Plasma HDL cholesterol (*p* < 0.0001) ↓ Plasma cholesterol/HDL cholesterol ratio (*p* = 0.006) ↑ LDL particle size (+0.7 Å; *p* < 0.001) ↓ The proportion of small dense LDL (−3.2%; *p* < 0.01) ↑ LDL ApoB100 fractional catabolic rate (+11.4%; *p* = 0.008) and the production rate (+9.4%; *p* = 0.03). ↑ Proportion of responders where plasma triglyceride concentrations reduced by >0·25 mmoL/L (45 vs. 32%, *p* < 0·001).
Klingel et al. (45)	Healthy men and women	Olive oil	No effect on serum triglycerides, total and HDL cholesterol ↑ Lipogenic index and *de novo* lipogenesis Trend for ↑ serum LPL activity	↓ Serum triglycerides No effect on serum total and HDL cholesterol No effect on lipogenic index or *de novo* lipogenesis ↑ Serum LPL activity	N/A
Pisaniello et al. (49)	Healthy adults	Mixed oils	No effect on serum total, HDL or LDL cholesterol or triglycerides	No effect on serum total, HDL or LDL cholesterol ↓ Serum triglycerides by an average of 0.31 mmoL/L (−27%, *p* = 0.02)	No differences
So et al. (48)	Older men and postmenopausal women with chronic inflammation	Sunflower oil	↓ Plasma triglycerides No effect on plasma total, HDL, LDL and non-HDL cholesterol, ApoA1 and ApoB	↓ Plasma triglycerides ↑ Plasma LDL No effect on plasma total, HDL and non-HDL cholesterol, ApoA1 and ApoB	No differences

Apo, apolipoprotein; HDL, high density lipoprotein; LDL, low density lipoprotein; LPL, lipoprotein lipase; PCSK9, proprotein convertase subtilisin/kexin type 9; VLDL, very low density lipoprotein. N/A indicates that a statistical comparison between the effects of EPA and DHA was not made.

The study of Grimsgaard et al. (19) in healthy men, found that both EPA (3.8 g/day) and DHA (3.6 g/day) for 7 weeks led to significant reductions in triglycerides (21 and 26%, respectively) compared to corn oil. Moreover, EPA demonstrated additional benefits by lowering total cholesterol, ApoA1 and ApoB, while DHA increased HDL cholesterol. Both EPA and DHA decreased the ratio of total to HDL cholesterol. Compared to EPA, DHA increased HDL cholesterol and showed a greater decrease in triglycerides, although the latter effect was not statistically significant.

The Mori et al. (23) study involving overweight mildly hyperlipidaemic men using 3.8 g/day of EPA or 3.7 g/day of DHA for 6 weeks demonstrated a decrease in triglycerides for both EPA and DHA (−18% and − 20%, respectively) compared to olive oil. EPA also decreased the HDL3 cholesterol subfraction by 7%, while DHA increased LDL cholesterol by 8%, HDL2 cholesterol by 29% and LDL particle size. EPA did not affect total, LDL, HDL or HDL2 cholesterol or LDL particle size, while DHA did not affect total, HDL or HDL3 cholesterol.

In the Woodman et al. (26, 28) study on hypertensive diabetics, EPA (3.8 g/day) or DHA (3.7 g/day) for 6 weeks had no impact on total, LDL or HDL cholesterol. However, there was a significant decrease in triglycerides with both EPA and DHA (−19% and − 15%, respectively) and there was an elevation in HDL2 (+16% and + 12%, respectively). EPA decreased HDL3 (−11%) but DHA had no effect. DHA but not EPA increased LDL particle size.

In the Nestel et al. (30) study involving dyslipidaemic patients that lasted 7 weeks, there was no observed change in total, LDL or HDL cholesterol levels with either EPA or DHA (3.0 and 2.8 g/day, respectively) compared to olive oil. However, both EPA and DHA were found to lower triglycerides (−23% and − 32%, respectively) and VLDL triglycerides, although there was no significant difference between the effects of the two fatty acids.

Surprisingly, in the Park and Harris study (32, 33), neither EPA nor DHA, at a dosage of 3.8 g/day for 4 weeks, had an impact on triglycerides or on total, LDL, HDL or VLDL cholesterol. This may be because of the short duration of this trial. Nevertheless, there was a significant reduction in ApoB48 and ApoB100 after a high fat challenge, suggesting more efficient handling of dietary fat. In agreement with this, both EPA and DHA increased chylomicron clearance (shorter half-life) and decreased chylomicron particle size when compared to safflower oil. Furthermore, lipoprotein lipase (LPL) activity increased following supplementation of either EPA or DHA, perhaps explaining the faster clearance of chylomicrons. The margination volume, which indicates the extent to which triglyceride-rich lipoproteins adhere to endothelial LPL, was increased with both EPA and DHA (+64% and + 53%, respectively) in the fasted state and with DHA in the fed state, consistent with more rapid triglyceride clearance.

The Allaire et al. (34, 43, 44) study conducted in healthy subjects with subclinical inflammation and abdominal obesity, showed that both EPA and DHA at 2.7 g/day for 10 weeks lowered triglycerides (−12% and − 13%, respectively), and increased LDL cholesterol (+2% and + 7%, respectively), compared to corn oil. The proportion of responders where there was a reduction in plasma triglyceride concentrations by >0.25 mmoL/L was greater with DHA than EPA (45 and 32%, respectively), although the average magnitude of triglyceride reduction was similar between DHA and EPA (0.59 and 0.57 mmol/L, respectively). Compared to control, DHA increased total cholesterol by 4%, increased HDL cholesterol by 8%, lowered the cholesterol/HDL ratio by 3% and increased ApoB by 5%. EPA did not have those effects. Compared to EPA, DHA resulted in a decrease in triglycerides, an increase in total cholesterol and LDL cholesterol (this was more prominent in men than women), an increase in HDL cholesterol and a decrease in the cholesterol/HDL cholesterol ratio. EPA decreased mean LDL particle size, while DHA increased it. Compared to EPA, DHA increased mean LDL particle size and decreased the proportion of small dense LDL by 3.2%. EPA and DHA both decreased PCSK9 concentrations (18.2 and 25% respectively). Furthermore, compared to EPA, DHA increased both the LDL ApoB100 production rate and the fractional catabolic rate.

Klingel et al. (45), found that DHA (3 g/day for 12 weeks) decreased serum triglycerides (from 0.85 ± 0.04 mmol/L to 0.65 ± 0.03 mmol/L) in healthy adults but EPA at the same dose did not. Neither EPA nor DHA significantly affected total or HDL cholesterol in this trial. Both EPA and DHA led to similar increases in serum LPL activity (from 44.1 to 49.1 mU/ml and from 42.9 to 51.5 mU/ml). In another trial involving healthy adults (49), DHA (4 g/day) was found to decrease triglycerides by an average of 27%, but there was no effect on total, LDL or HDL cholesterol or effect of EPA on blood lipids. Overall, there was no difference in effects of EPA and DHA on blood lipids including triglycerides. The duration of this study was only 4 weeks, which is shorter than other trials that show greater effects.

So et al. (48) found that both EPA and DHA (3.0 g/day for 10 weeks) decreased plasma triglyceride concentrations (−20% and −22%, respectively) in older adults with inflammation without changes in ApoB concentrations, resulting in a significant reduction in triglyceride/ApoB ratio with both EPA and DHA. EPA did not affect total, HDL, LDL or non-HDL cholesterol, while DHA raised LDL cholesterol. The LDL-C/ApoB and HDL-C/ApoA1 ratios were increased with both EPA and DHA but the increase of the HDL-C/ApoA1 ratio was greater with DHA. This study also reported the effects of EPA and DHA on the activities of LPL, cholesteryl ester transfer protein (CETP) and lecithin cholesterol acyl transferase (LCAT). These were all affected by both EPA and DHA but in a sex-specific way. DHA increased LPL activity and decreased LCAT activity with the latter only seen in women. EPA decreased CETP and LCAT activity with the decrease in LCAT again seen only in women.

#### Effect of EPA vs. DHA on inflammatory markers

Four trials (five publications) (29, 34, 36, 47, 49) included outcomes related to the effect of EPA and DHA on inflammatory markers (Table 5). The Allaire et al. (34)/Vors et al. (36) study observed a significant reduction in plasma levels of CRP, IL-6, IL-18 and TNF-*α* (−8, −12, −7, and − 15% respectively) and an increase in adiponectin (3%) with DHA supplementation (2.7 g/day) compared to corn oil. In contrast, EPA at the same dose only decreased plasma IL-6 (−13%). Compared to EPA, DHA resulted in a greater decrease in IL-18 and a greater increase in adiponectin. Both EPA and DHA decreased CD14 gene expression and increased PPARA gene expression. EPA also increased expression of the TRAF3 gene, while DHA increased expression of the TNFA gene. In contrast to some of these findings, Mori et al. (29) saw no effect of EPA (3.8 g/day) or DHA (3.7 g/day) on plasma CRP or IL-6, while both EPA and DHA tended to decrease plasma TNF-*α*, with DHA having a greater effect, although this was not formally tested statistically. So et al. (47) reported no effect of EPA or DHA (3 g/day) on plasma CRP, TNF-α, IL-6, MCP-1 or IL-10 and no difference in effect of EPA and DHA on these biomarkers of inflammation. DHA decreased monocyte secretion of TNF-α, IL-6 and MCP-1 in response to lipopolysaccharide and decreased expression of the TNFA, IL6, MCP1 and IL10 genes. EPA only decreased expression of the TNFA gene. Both EPA and DHA modified the plasma oxylipin profile; both decreased several arachidonic acid (AA)-derived oxylipins. EPA increased several EPA-derived oxylipins while DHA increased several DHA- and EPA-derived oxylipins. Finally, Pisaniello et al. (49) reported no effect of EPA or DHA (4 g/day) on serum CRP in healthy adults. Serum from those supplemented with EPA decreased CCL2 gene expression in endothelial cells. Other genes were unaffected and serum from those supplemented with DHA had no effect on any genes.

**Table 5:** Summary of findings related to effects of EPA and DHA on inflammatory markers.

Study	Population	Control	Effect of EPA vs. control	Effect of DHA vs. control	Effect of EPA vs. DHA
Mori et al. (29)	People with type-2 diabetes and treated for hypertension	Olive oil	No effect on plasma IL-6 and CRP Trend for ↓ plasma TNF-α (−19.5%, n.s.)	No effect on plasma IL-6 and CRP Trend for ↓ plasma TNF-α (−32.8%, n.s.)	N/A
Allaire et al. (34), Vors et al. (36)	Healthy subjects with abdominal obesity and low-grade inflammation	Corn oil	No effect on plasma CRP, IL-18, TNF-α or adiponectin ↓ Plasma IL-6 (−13%, *p* = 0.03) ↓ CD14 gene expression (*p* = 0.008) ↑ PPARA (*p* = 0.003) and TRAF3 gene expression (*p* = 0.002)	↓ Plasma CRP (−8%, *p* = 0.02), TNF-α (−15%, *p* = 0.01), IL-6 (−12%, *p* = 0.01) and IL-18 (−7%, *p* = 0.002) ↑ Plasma adiponectin (+3%, *p* = 0.047) ↓ CD14 gene expression (*p* = 0.02) ↑ PPARA (*p* = 0.01) and TNFA gene expression (*p* = 0.01)	Compared to EPA, DHA ↓ IL-18 (*p* = 0.01) and ↑ adiponectin (<0.001) No difference for CRP, IL-6, and TNF-α or for gene expression
Pisaniello et al. (49)	Healthy adults	Mixed oils	No effect on serum CRP Serum from EPA supplementation ↓ CCL2 gene expression by endothelial cells (−25%, *p* = 0.03); other genes unaffected	No effect on serum CRP No effect of serum from DHA supplementation on inflammatory gene expression by endothelial cells	N/A
So et al. (47)	Older men and postmenopausal women with chronic inflammation	Sunflower oil	No effect on plasma CRP, TNF-α, IL-6, MCP-1 or IL-10 ↓ LPS-induced monocyte TNFA gene expression but no effect on IL6, MCP1 or IL10 ↓ ratio of LPS induced monocyte TNF to IL10 and MCP1 to IL10 gene expression ↑ plasma EPA-derived oxylipins	No effect on plasma CRP, TNF-α, IL-6, MCP-1 or IL-10 ↓ LPS induced monocyte secretion of TNF-α, IL-6 and MCP-1 ↓ LPS induced monocyte TNFA, IL6, MCP1 and IL10 gene expression ↑ plasma DHA-derived oxylipins	No difference for plasma CRP, TNF-α, IL-6, MCP-1 or IL-10 DHA ↓ LPS induced monocyte IL10 gene expression compared with EPA Different plasma oxylipin profiles

CCL, chemokine (C-C motif), ligand; CRP, C-reactive protein; IL, interleukin; LPS, lipopolysacharide; MCP, monocyte chemoattractant protein; PPAR, peroxisome proliferator activated receptor; TNF, tumor necrosis factor; TRAF, tumor necrosis factor receptor associated factor. N/A indicates that a statistical comparison between the effects of EPA and DHA was not made.

#### Effect of EPA vs. DHA on blood pressure, haemodynamics, and vascular function

Six trials (eight publications) (20–22, 26, 27, 30, 46, 49) included outcomes related to effects of EPA and DHA on blood pressure, haemodynamics and vascular function (Table 6). Grimsgaard et al. (20) reported an increase in heart rate (+1.9 bpm) with EPA (3.8 g/day) but a decrease in heart rate (−2.2 bpm) with DHA (3.6 g/day) in healthy men. When directly compared with each other, DHA resulted in a decreased heart rate compared to EPA. Both EPA and DHA improved left ventricular diastolic filling but there was found to be no significant effect of either EPA or DHA on blood pressure. In the Mori et al. (21, 22) study in overweight mildly hyperlipidaemic men, DHA (3.7 g/day) decreased both systolic and diastolic blood pressure compared to placebo. However, EPA (3.8 g/day) was found to have no significant effect on blood pressure. DHA decreased heart rate by around 3.5 bpm over a 24-h period compared to placebo. Furthermore, DHA increased vasodilator responses and attenuated constrictor responses in forearm blood flow compared to placebo. In the Nestel et al. (30) study in dyslipidaemic subjects, there was no effect of EPA (3 g/day) or DHA (2.8 g/day) on heart rate or blood pressure. However, there was an increase in systemic arterial compliance with EPA (+36%) and DHA (+27%) compared to placebo. There was also a non-significant lowering of vascular resistance with both EPA and DHA. There was no significant difference between the effects of EPA and DHA for any of the parameters. In the study of anti-hypertensive-treated type 2 diabetics by Woodman et al. (26, 27), there was no significant effect of EPA (3.8 g/day) or DHA (3.7 g/day) on blood pressure or vascular function. However, there was a non-significant decrease in 24 h heart rate with both EPA and DHA. In a study of healthy young men and women, Lee et al. (46) reported an increase in heart rate (4.2 beats/min) and systolic and diastolic blood pressure with EPA compared to the olive oil placebo, but no effect of DHA (both at 3 g/day); effects of EPA were different from those of DHA. DHA, but not EPA, increased cardiac muscle sympathetic nerve activity burst frequency and incidence. Finally, Pisaniello et al. (49) reported no effect of EPA (4 g/day) on heart rate or blood pressure in heathy adults. DHA (4 g/day) also did not affect heart rate or systolic blood pressure, but decreased diastolic blood pressure.

**Table 6:** Summary of findings related to effect of EPA and DHA on blood pressure, haemodynamics and vascular function.

Study	Population	Control	Effect of EPA vs. control	Effect of DHA vs. control	Effect of EPA vs. DHA
Grimsgaard et al. (20)	Healthy adult men	Corn oil	↑ Heart rate (increased 1.9 bpm, *p* = 0.04) No effect on systolic or diastolic blood pressure Improved left ventricular diastolic filling	↓ Heart rate (decreased 2.2 bpm, *p* = 0.006) No effect on systolic or diastolic blood pressure Improved left ventricular diastolic filling	DHA ↓ heart rate compared with EPA (*p* = 0.0001)
Mori et al. (21, 22)	Overweight mildly hyperlipidaemic men	Olive oil	No significant effect on blood pressure Small nonsignificant rise in heart rate No effect on vasodilator or constrictor responses of forearm blood flow	↓ 24 h systolic (−5.8 mmHg, p = 0.022) and diastolic (−3.3 mmHg, *p* = 0.029) blood pressure ↓ daytime systolic (−3.5 mmHg, *p* = 0.041) and diastolic (−2.0 mmHg, p = 0.046) blood pressure ↓ 24 h (−3.5 bpm, p = 0.001), daytime (−3.7 bpm, p = 0.001), night-time (−2.8 bpm, *p* = 0.025) ambulatory heart rate ↑ vasodilator responses and ↓ constrictor responses of forearm blood flow	N/A
Woodman et al. (26, 27)	People with type-2 diabetes treated for hypertension	Olive oil	No effect on systolic or diastolic blood pressure Nonsignificant decrease in 24 h heart rate No effect on flow-mediated dilatation or glyceryl-trinitrate mediated dilatation	No effect on systolic or diastolic blood pressure Nonsignificant decrease in 24 h heart rate No effect on flow-mediated dilatation or glyceryl-trinitrate mediated dilatation	N/A
Nestel et al. (30)	Dyslipidaemic subjects	Olive Oil	No effect on heart rate No effect of systolic or diastolic blood pressure No effect on pulse pressure ↑ Systemic arterial compliance (+36%, *p* = 0.028) Trend to decrease total vascular resistance	No effect on heart rate No effect of systolic or diastolic blood pressure No effect on pulse pressure ↑ Systemic arterial compliance (+27%, *p* = 0.091) Trend to decrease total vascular resistance	No differences between EPA and DHA
Lee et al. (46)	Healthy young adults	Olive Oil	↑ Heart rate (4.2 bpm, *p* = 0.04) ↑ Systolic blood pressure (*p* = 0.01) ↑ Diastolic blood pressure (*p* = 0.002) No effect on cardiac muscle sympathetic nerve activity burst frequency and burst incidence	No effect on heart rate No effect on systolic or diastolic blood pressure ↑ Cardiac muscle sympathetic nerve activity burst frequency (*p* = 0.001) and burst incidence (*p* = 0.003)	EPA ↑ heart rate compared with DHA (p = 0.05) EPA ↑ systolic blood pressure compared with DHA (*p* = 0.008) EPA ↑ diastolic blood pressure compared with DHA (*p* = 0.04) DHA ↑ cardiac muscle sympathetic nerve activity burst frequency (p = 0.02) and burst incidence (*p* = 0.058) compared to EPA
Pisaniello et al. (49)	Healthy adults	Mixed oils (palm oil, sunflower oil, rapeseed oil, and fish oil)	No effect on heart rate No effect on systolic blood pressure No effect on diastolic blood pressure	No effect on heart rate No effect on systolic blood pressure ↓ Diastolic blood pressure (−4.1 ± 1.8 mmHg, p = 0.05)	N/A

N/A indicates that a statistical comparison between the effects of EPA and DHA was not made.

#### Effect of EPA vs. DHA on glycaemic control

Three trials (23, 26, 45) included outcomes related to effects of EPA and DHA on glycaemic control (Table 7). In the Mori et al. (23) trial in overweight mildly hyperlipidaemic men, fasting insulin was found to be increased by both EPA (+18%) and DHA (+27%). With EPA there was also a trend toward increased fasting glucose (+4%) but there was no effect of DHA on fasting glucose. With DHA, there was a decrease in the glucose:insulin ratio, as a result of the effect on insulin. In type-2 diabetics treated for hypertension (26), both EPA and DHA increased fasting glucose, with a larger effect of EPA than DHA (+19 vs. +12%). There was no effect of either EPA or DHA on fasting insulin, glycated hemoglobin, fasting C-peptide, insulin sensitivity or insulin secretion compared to control. Klingel et al. (45) reported no effect of either EPA or DHA (3 g/day) on fasting glucose compared to olive oil in healthy participants.

**Table 7:** Summary of findings related to effects of EPA and DHA on glycaemic control.

Study	Population	Control	Effect of EPA vs. Control	Effect of DHA vs. Control	Effect of EPA vs. DHA
Mori et al. (23)	Overweight mildly hyperlipidaemic men	Olive oil	Trend toward increased fasting glucose (+4%, *p* = 0.062) ↑ Fasting insulin (+18%, *p* = 0.035)	No effect on fasting glucose ↑ Fasting insulin (+27%, *p* = 0.001) ↓ Glucose to insulin ratio (*p* = 0.018)	N/A
Woodman et al. (26)	People with type-2 diabetes treated for hypertension	Olive oil	↑ Fasting glucose (+19%, *p* = 0.002) No effect on glycated hemoglobin, fasting insulin, fasting C-peptide, insulin sensitivity or secretion	↑ Fasting glucose (+12%, *p* = 0.002) No effect on glycated hemoglobin, fasting insulin, fasting C-peptide, insulin sensitivity or secretion	N/A
Klingel et al. (45)	Healthy participants	Olive oil	No effect on fasting glucose	No effect on fasting glucose	No effect on fasting glucose

N/A indicates that a statistical comparison between the effects of EPA and DHA was not made.

#### Effect of EPA vs. DHA on platelet and fibrinolytic function

Two trials (28, 31) included outcomes related to effects of EPA and DHA on platelet and fibrinolytic function (Table 8). The Park and Harris (31) trial conducted in healthy subjects reported a decrease in mean platelet volume and an increase in platelet numbers with EPA (3.8 g/day) compared with safflower oil. However, with DHA (3.8 g/day), there was no effect on platelet volume or count. The effects of EPA were significantly different from those of DHA. The effects on platelet aggregation were not assessed in that trial. In the Woodman et al. (28) trial in anti-hypertensive-treated type 2 diabetics DHA, in contrast to EPA, led to a decrease in collagen-stimulated platelet aggregation (−17%) and platelet-derived thromboxane B2 release (−19%) when compared to olive oil. Thrombaxane B2 is derived from thromboxane A2 which promotes platelet aggregation and so these two observations with DHA may be related. In that trial, neither EPA nor DHA demonstrated any effect on markers of fibrinolytic function.

**Table 8:** Summary of findings related to effect of EPA and DHA on platelet and fibrinolytic function.

Study	Population	Control	Effect of EPA vs. Control	Effect of DHA vs. Control	Effect of EPA vs. DHA
Park and Harris (31)	Healthy subjects	Safflower oil	↓ Mean platelet volume ↑ Platelet count	No effect	EPA ↓ Mean platelet volume EPA ↑ Platelet count
Woodman et al. (28)	People with type-2 diabetes treated for hypertension	Olive oil	Platelet function: No effect on collagen-or PAF-stimulated platelet aggregation or platelet-derived TXB_2_ Fibrinolytic function: No effect on PAI-1, tPA, von Willebrand factor, or P-selectin.	Platelet function: ↓ Collagen-stimulated platelet aggregation (−17%, *p* = 0.054) ↓ Platelet-derived TXB_2_ (−19%, *p* = 0.03) No effect on PAF-stimulated platelet aggregation Fibrinolytic function: No effect on PAI-1, tPA, von Willebrand factor, or P-selectin	N/A

PAF, platelet activating factor; PAI, plasminogen activator inhibitor; tPA, tissue plasminogen activator; TX, thromboxane. N/A indicates that a statistical comparison between the effects of EPA and DHA was not made.

#### Effect of EPA vs. DHA on oxidative stress

Two trials (three publications) (24, 25, 29) included outcomes related to effects of EPA and DHA on oxidative stress as assessed by measuring F2 isoprostances in urine and plasma (Table 9). In a trial in overweight mildly hyperlipidaemic men (24, 25), both EPA and DHA significantly decreased urinary F2 isoprostanes (by 27 and 26% respectively) and plasma F2 isoprostanes (by 24 and 14% respectively) compared to olive oil. Likewise, in a trial in type 2 diabetics treated for hypertension (25, 29), both EPA and DHA significantly decreased urinary F2 isoprostanes (by 19 and 20% respectively) and plasma F2 isoprostanes (by 19 and 23% respectively) compared to olive oil. The effects of EPA and DHA were not formally compared but DHA tended to have greater effects than EPA on F2 isoprostanes as a marker of oxidative stress.

**Table 9:** Summary of findings related to effect of EPA and DHA on oxidative stress.

Study	Population	Control	Effect of EPA vs. control	Effect of DHA vs. control	Effect of EPA vs. DHA
Mori et al. (24), Mas et al. (25)	Overweight mildly hyperlipidaemic men	Olive oil	↓ Urinary F_2_ isoprostanes (−27%, *p* < 0.0001) ↓ Plasma F_2_ isoprostanes (−24%, *p* < 0.0001)	↓ Urinary F_2_ isoprostanes (−26%, *p* < 0.0001) ↓ Plasma F_2_ isoprostanes (−14%, *p* = 0.009)	N/A
Mori et al. (29); Mas et al. (25)	People with type-2 diabetes treated for hypertension	Olive oil	↓ Urinary F_2_ isoprostanes (−19%, *p* = 0.017) ↓ Plasma F_2_ isoprostanes (−19%, *p* = 0.039)	↓ Urinary F_2_ isoprostanes (−20%, *p* = 0.014) ↓ Plasma F_2_ isoprostanes (−23%, *p* = 0.011)	N/A

N/A indicates that a statistical comparison between the effects of EPA and DHA was not made.

## Discussion

### Context of this systematic review

Higher dietary intakes and higher blood and tissue status of the omega-3 PUFAs EPA and DHA are associated with lower risk of developing CVD and mortality from CVD, including coronary heart disease (3, 4) and some studies have reported that intervention with these fatty acids decreases adverse cardiovascular outcomes in at risk patients (50, 51). EPA and DHA act through beneficial effects on multiple cardiovascular risk factors, as demonstrated in numerous trials and meta-analyses of RCTs (2–4, 6–16). An important question that is not fully resolved is whether EPA and DHA have the same or unique actions (52, 53). A systematic review of RCTs that compared the effect of ≥2 g/day of near pure EPA and DHA on cardiovascular risk factors was published in 2018 (18). It included 18 publications from 6 unique RCTs and concluded that EPA and DHA do appear to have differential effects on at least some risk factors for CVD. New information on this topic has been published since mid-2017 when the literature search for that systematic review was conducted. Therefore, the search was updated and this systematic review produced. The new search identified an additional seven publications including five publications from 3 new RCTs (45–49) and 2 from one of the previously included RCTs (43, 44). New data on plasma lipids and lipoproteins (43–45, 47, 48), inflammatory biomarkers (47, 49), blood pressure, haemodynamics and vascular function (46, 49) and glycaemic control (45) are included.

### Summary of effects of EPA vs. DHA on cardiovascular risk factors

Six out of nine trials reported that EPA lowered triglycerides compared with placebo, while seven out of nine trials reported that DHA lowered triglycerides. Although some trials suggest a greater triglyceride lowering effect of DHA than EPA, the difference sometimes appears to be small, and is not apparent in some trials. Effects of EPA and DHA on cholesterol, LDL and HDL are less consistent. Most trials report no effect of EPA on LDL cholesterol, although one reported an increase (34). Although some trials report no effect of DHA on LDL cholesterol, 3 reported an increase (23, 34, 48). No included trials reported that EPA affects HDL cholesterol and, while most trials also report no effect of DHA, two did report increased HDL cholesterol with DHA (19, 34). There may be effects of EPA and DHA on HDL subfractions: two trials reported that EPA lowers HDL3 (26, 34) while one reported higher HDL2 (26). Two trials reported that DHA increases HDL2 (23, 26). Regarding LDL particle size, two trials reported no effect of EPA (23, 28), while one reported a decrease in size (43). Three trials reported that DHA increased LDL particle size (23, 28, 43). The trials of Park and Harris (32, 33) and So et al. (48) indicate that EPA and DHA can have effects on the enzymes that metabolize lipoproteins and control the transfer of lipid moieties between lipoproteins. Thus, high dose EPA and DHA lower triglycerides, with DHA possibly being more potent. EPA has little impact on either LDL or HDL cholesterol, but may lower the level of the harmful HDL3 subfraction. DHA can raise both LDL and HDL cholesterol, may raise the level of the protective HDL2 subfraction, and increases LDL particle size, perhaps rendering LDL less atherogenic.

Regarding inflammatory markers, all four included trials reported that EPA did not alter CRP levels; 3 out of the 4 trials also report no effect of DHA, but one (34) reported that DHA lowered CRP. No included trial reported that EPA altered any of the cytokines assessed (TNF-*α*, IL-6, IL-10, MCP-1, IL-18, adiponectin), but these were mostly only measured in one trial. One trial did report a trend to lower TNF-α with EPA (29). The trial that reported that DHA lowered CRP also found lower TNF-α, IL-6 and IL-18 and higher adiponectin with DHA (34). Another trial (29) reported a trend to lower TNF-α with DHA. Both EPA and DHA decreased CD14 gene expression and increased PPARA gene expression in one trial (36). Both EPA and DHA decreased the inflammatory response seen with LPS stimulation of monocytes studied *ex vivo*, with a stronger effect of DHA (47). Both EPA and DHA modified the plasma oxylipin profile (47) Thus, both EPA and DHA promoted an anti-inflammatory gene expression profile and reduced the response of monocytes to an inflammatory stimulus; however circulating biomarkers of inflammation like CRP and IL-6 are little impacted by EPA, but these may be lowered by DHA suggesting that DHA has stronger anti-inflammatory actions. Both EPA and DHA foster a less inflammatory plasma profile of oxylipins.

Five out of 6 trials reported no effect of EPA on blood pressure, 4 out of 6 reported no effect on heart rate and 2 out of 3 reported no effect on vascular function. One trial reported that EPA increased systolic and diastolic blood pressure in healthy young adults compared to the olive oil placebo (46). Two trials reported that EPA increased heart rate in healthy adult men (20, 46), while another reported a trend to decreased heart rate in people with diabetes being treated for hypertension (26). One trial reported that EPA improved arterial compliance in people with dyslipidaemia (30). Four out of 6 trials reported no effect of DHA on blood pressure, 3 out of 6 reported no effect on heart rate and 1 out of 3 reported no effect on vascular function. One trial reported that DHA decreased systolic and diastolic blood pressure in overweight mildly hyperlipidaemic men (21) while another reported lower diastolic, but not systolic, blood pressure in healthy adults (49). Two trials reported that DHA lowered heart rate (20, 21), while a third trial reported a trend for this (26). Two trials reported that DHA improved vascular function (22, 30). Overall, DHA appears to have stronger and more favorable effects on blood pressure, heart rate and vascular function than EPA.

One trial reported that EPA increased fasting glucose (26); another reported a trend for this outcome (23) but a third found no effect of EPA (45). One trial reported that EPA increased fasting insulin (23) but a second trial did not see this (26). That trial found no other effects of EPA on markers of glucose homeostasis including insulin sensitivity. One trial reported that DHA increased fasting glucose (26); two others found no effect of DHA (23, 45). One trial reported that DHA increased fasting insulin (23) but a second trial did not see this (26). That trial found no other effects of DHA on markers of glucose homeostasis including insulin sensitivity. Overall, there seems to be little impact of EPA and DHA on glucose homeostasis.

One trial reported that EPA decreases mean platelet volume and increases platelet number, with DHA not having these effects (31). A second trial reported that DHA decreased collagen-stimulated platelet aggregation and thromboxane B2 generation, but EPA did not have these effects (28). There was no effect of EPA or DHA on markers of fibrinolytic function in the one trial that reported these (28).

Both EPA and DHA were found to decrease urinary and plasma F2 isoprostanes assessed as markers of oxidative stress, with little difference in potency (24, 25, 29).

## Discussion of the findings

This systematic review suggests that EPA and DHA have quantitatively different effects on CVD risk factors such as blood lipids including triglycerides, blood pressure, heart rate, vascular function, platelet function, and inflammatory markers. The trials included in this review show that DHA has a more favorable impact on several of these parameters.

It is suggested that the plasma triglyceride lowering effect of both EPA and DHA is due to a number of factors, including an inhibitory effect on hepatic triglyceride synthesis and VLDL assembly and secretion and an enhanced triglyceride hydrolysis and clearance of VLDL by LPL (54). This was supported by the Klingel et al. (45) trial that showed that both EPA and DHA increased LPL activity. DHA had a greater effect on LPL activity, which might account for its (slightly) greater triglyceride lowering action. The decrease in hepatic triglyceride synthesis and VLDL secretion is thought to be due to the decrease in synthesis of ApoB100, which is required for VLDL assembly. A decrease in hepatic VLDL assembly and secretion is thought to be beneficial for lowering blood triglyceride levels, as VLDL is the main lipoprotein that transports triglycerides (54). Interestingly, Allaire et al. (44) reported an increase in the VLDL ApoB100 catabolism rate which is the rate at which VLDL is cleared from the bloodstream, which further supports the decrease in triglyceride levels observed with EPA and DHA. VLDL catabolism generates LDL, so this could be part of the mechanism for the elevated LDL-cholesterol reported in some studies of DHA.

In terms of the results with other blood lipids and blood lipid-related factors, one of the newer trials identified a decrease in PCSK9 levels with both EPA and DHA (43). PCSK9 degrades the cell surface LDL receptors responsible for clearing circulating LDL and therefore contributes to elevated LDL-cholesterol (55). An EPA- or DHA-mediated decrease in PCSK9 would be anticipated to result in better LDL clearance and so lower LDL-cholesterol. Contrary to this expectation, some trials report elevated LDL-cholesterol, especially with DHA (23, 34, 48). Apart from an increase in LDL levels, some trials reported an increase in LDL particle size with DHA supplementation (23, 28, 43). Allaire et al. (43) suggest that the reason for this could be due to the decrease in ApoCIII secretion from the liver through the regulation of transcriptions factors and binding proteins. This apparent reduction in ApoCIII production after DHA leads to increased conversion of VLDL to LDL and the formation of larger LDL particles compared to EPA, as reported in several trials. In this regard DHA also decreased the proportion of pro-atherogenic small dense LDL particles (43). The increase in HDL cholesterol that is reported with DHA could be explained by altered activity of lipid transfer proteins such as CETP (48) which results in the transfer of cholesteryl esters from HDL toward more triglyceride-rich lipoproteins.

Regarding the effects of EPA and DHA on inflammation, there is some inconsistency in the findings of the included trials. In terms of their actions on inflammation, EPA and DHA are thought to have anti-inflammatory effects through the replacement of, and competition with, AA in the cell membrane which results in decreased production of eicosanoids from AA which tend to be more inflammatory (such as prostaglandin E2 and leukotriene B4) and increased production of eicosanoids from EPA (leukotriene B5 and prostaglandin E3) which are less inflammatory (56, 57). Furthermore, EPA and DHA both give rise to lipid mediators termed specialized pro-resolving mediators (58–60). Interestingly, So et al. (47) reported a greater reduction in plasma phospholipid AA with DHA than EPA linked with a greater reduction in AA derivatives such as prostaglandin E2 and thromboxane B2. There was also an elevation in some of the EPA- and DHA-derived oxylipins (47). EPA and DHA are also known to have a role in regulating inflammatory gene expression (56, 57), effects seen in the studies reported in Allaire et al. (34, 36) and So et al. (47). Markers of inflammation such as CRP, IL-6, IL-1, and TNF-*α* are linked with an increased likelihood of CVD and cardiovascular events (61, 62). Furthermore, there is a relationship between the inflammatory markers themselves, for example IL-6 triggers CRP to be synthesized in the liver. Despite the reported effects on oxylipins and gene expression in the included trials (34, 36, 47), there were few effects on circulating markers of inflammation, although Allaire et al. (34) reported a decrease in plasma CRP, TNF-α, IL-6, and IL-18 with DHA and a decrease in IL-6 with EPA.

With respect to the limited trials that show a decrease in blood pressure with EPA and DHA, it is suggested that the mechanism for this is related to a decrease in systemic vascular resistance due to the increase in nitric oxide production (63), decreased response to angiotensin II and noradrenalin (64, 65) and an increase in arterial compliance (66) leading to a reduction in systolic and diastolic pressure. The changes in heart rate observed with DHA can be explained by the beneficial impacts on cardiac muscle cell function and the likely changes in membrane fluidity, which alter the conductive properties of ion channels within those membranes (67) resulting in lowered heart rate.

Regarding glycaemic control, the results are limited, but some trials report that EPA and DHA may increase fasting glucose and fasting insulin, which may be seen as a deleterious effect. These effects may be due to an increase in hepatic glucose output and an increase in plasma glucagon concentrations. It is worth noting that the recent trial by Klingel et al. (45) reported no effect of either EPA or DHA on blood glucose.

EPA and DHA appear to have different effects on platelet function with EPA reducing mean platelet volume and DHA reducing collagen-induced platelet aggregation. The mechanism behind effects on platelet aggregation is similar to the effects on inflammation, in that EPA and DHA displace AA from the platelet membrane leading to a decrease in thromboxane A2 which is a platelet aggregator and an increase in EPA-derived prostacyclin PGI3 which is an inhibitor of platelet aggregation (68). Regarding platelet volume, an increase in platelet volume would suggest a more pro-atherogenic environment; therefore, EPA may reduce the incidence of atherogenic events via the reduction of mean platelet volume; DHA seems not to have this effect.

In the context of oxidative stress, both plasma and urinary F2-isoprostanes have been established as biomarkers indicative of *in vivo* lipid peroxidative damage (69). AA is the precursor for the synthesis of F2-isoprostanes (69). The reduction in F2-isoprostanes by EPA and DHA is interpreted to indicate reduced oxidative stress. However, since AA is the precursor to F2-isoprostanes, it may be that lower F2-isoprostanes also partly reflect the lowering of AA that is a feature of increased intake of EPA and DHA. Nevertheless, Mas et al. (25) reported that the effects of EPA and DHA on plasma F2-isoprostanes are retained when the data are adjusted for the change in plasma AA.

### The broader context

Irrespective of whether there are differences in their quantitative effects, the beneficial impact of both EPA and DHA on a range of recognized and emerging risk factors for CVD suggests that they play an important role in disease prevention. This is supported by multiple epidemiological studies which evaluate the association between intake or blood or tissue levels of EPA and DHA and incident disease during a, usually long, follow-up period. Chowdhury et al. (70) aggregated such prospective studies investigating risk of coronary outcomes. Data from 16 studies, including over 422,000 subjects, showed a 13% reduction in risk for those in the top third of dietary intake of EPA + DHA than those in the lower third of intake. Data from 13 studies with over 20,000 participants showed a 22, 21, and 25% reduction in risk of coronary outcomes for those in the top third of blood levels of EPA, DHA, or EPA + DHA, respectively, compared to those in the lower third (70). Using data from 17 prospective cohort studies, Alexander et al. (71) reported an 18% lower risk for any coronary heart disease event for subjects with higher dietary intake of EPA + DHA than for those with lower intake. There were also significant reductions of 19, 23, and 47% in the risk for fatal coronary death, coronary events, and sudden cardiac death, respectively. Another study pooled data from 19 trials investigating the association between EPA or DHA concentration in a body pool, such as serum, plasma, red blood cells, or adipose tissue, and risk of future coronary heart disease in adults who were healthy at study entry (72). Both EPA and DHA were independently associated with a reduction in the risk of fatal coronary heart disease, with about a 10% reduced risk for each one standard-deviation increase in either EPA or DHA. Harris et al. (73) gathered together 10 cohort studies and found a 15% lower risk of fatal coronary heart disease for each one-standard-deviation increase in the omega-3 index (i.e., the sum of EPA + DHA in red blood cells). A *de novo* pooled analysis of 17 prospective cohort studies with 42,466 individuals confirmed the association between a lower risk for death from CVD in those with the highest vs. the lowest quintile of circulating EPA, DHA, and EPA + DHA (74). These analyses support a clear role for EPA and DHA in the primary prevention of coronary heart disease and, perhaps more widely, of CVD, as discussed elsewhere (3, 4), findings that underpin current dietary recommendations for intake of these fatty acids (75–78). The Vitamin D and Omega-3 (VITAL) trial also provides some support for these recommendations. This was an RCT conducted as a two-by-two factorial design of vitamin D3 (at a dose of 50 μg/day) and EPA + DHA (1 g/day) among 25,871 healthy participants aged over 50 years for the primary prevention of CVD and cancer (79). After a median follow-up of 5.3 years, there was no statistically significant difference between the groups receiving EPA + DHA or placebo in the primary outcome of major cardiovascular events (a composite of myocardial infarction, stroke, or death from cardiovascular causes). However, an analysis of the individual components of the composite showed a significant reduction in the EPA + DHA arm for myocardial infarction (28% reduction) and coronary heart disease (17% reduction). Correspondingly, there was also a lower risk of death from these two non-prespecified outcomes (50% for myocardial infarction and 24% for coronary heart disease), although the effect on coronary heart disease mortality was not significant. There was a significant reduction in major adverse cardiovascular events (19%) and risk of myocardial infarction (40%) for those who consumed fewer than 1.5 fish meals per week and then supplemented with EPA + DHA. Although there was no effect on the primary outcome (first serious vascular event) between EPA + DHA (840 mg/day) and placebo groups over a median follow-up on 7.4 years in A Study of Cardiovascular Events in Diabetes (ASCEND), a study of 15,480 people living with diabetes but with no evidence of CVD, there were 19% fewer deaths from vascular events in the EPA + DHA arm as well as a trend toward reduced risk of death (21%) from coronary heart disease (80).

Despite the consistent evidence for EPA and DHA reducing risk of CVD, findings from trials using these fatty acids therapeutically in those already with high risk or with advanced disease have proven to be inconsistent (3, 4). This has led to discussion about the relative impact of EPA and DHA, since different therapeutic trials have used different formulations. In the GISSI-Prevenzione study (81) involving survivors of recent myocardial infarction (≤ 3 months since myocardial infarction), treatment with EPA + DHA (840 mg/day) significantly reduced the composite primary outcomes (−15% and − 20%, respectively) and several secondary outcomes, including cardiovascular death by 30%, sudden death by 45%, and total fatal events by 20%. In the GISSI-HF trial (82), patients with chronic heart failure received EPA + DHA (840 mg/day) or placebo for approximately 4 years, and there a small (9%) but significant reduction in all-cause mortality. These trials suggest that the combination of EPA + DHA may be effective therapeutically. The randomized, open-label Japan EPA Lipid Intervention Study (JELIS) included patients with hypercholesterolemia who were assigned to receive either a statin alone or a statin along with highly purified EPA (1.8 g/day EPA) with a 5-year follow-up (83). The primary outcome was any major coronary event, including sudden cardiac death, fatal and nonfatal myocardial infarction, and other nonfatal events, including unstable angina pectoris, angioplasty, stenting, and coronary artery bypass grafting. Long-term use of EPA-ethyl ester as an addition to statin therapy had no effect over statin alone on the primary outcome in the primary prevention arm of the trial, but in the secondary prevention arm, EPA supplementation resulted in a significant 19% reduction in nonfatal coronary events vs. statin alone (83). JELIS highlights that EPA may be effective in the absence of DHA. This latter conclusion is supported by more recent trials. In the Reduction of Cardiovascular Events with Icosapent Ethyl Intervention Trial (REDUCE-IT) (50), 8,179 high risk patients received 3.6 g/day of EPA as ethyl ester or mineral oil as placebo with a median follow-up of 4.9 years. The primary outcome (a composite of cardiovascular death, nonfatal myocardial infarction, nonfatal stroke, coronary revascularization, or unstable angina) was reduced by 25% in patients who received EPA-ethyl ester compared to placebo. The key prespecified secondary outcome (a composite of cardiovascular death, nonfatal MI, or nonfatal stroke) was also significantly reduced in the EPA-ethyl ester group as were a whole range of other clinical outcomes (50). Another positive EPA-ethyl ester trial was Effect of Vascepa on Improving Coronary Atherosclerosis in People with High Triglycerides Taking Statin Therapy (EVAPORATE) (84). This study involved 80 patients with known angiographic coronary artery disease taking statins and with no history of myocardial infarction, stroke, or life-threatening arrhythmia within the prior 6 months. The same EPA-ethyl ester preparation and the same dose were used as in REDUCE-IT. EVAPORATE demonstrated that EPA might directly promote atherosclerotic plaque attenuation in hypertriglyceridemic individuals at 18 months (84). In contrast to this series of trials demonstrating significant therapeutic benefit of EPA provided in the absence of DHA (50, 83, 84), trials of the combination of EPA + DHA conducted since the two GISSI trials provide inconsistent findings. The Outcome Reduction with an Initial Glargine Intervention (ORIGIN) trial with 840 mg/day EPA + DHA in 12,536 dysglycemic patients with recent myocardial infarction or heart failure and a median follow-up of 6.2 years was null (85), as was the Risk and Prevention Study which assessed the effect of 840 mg/day EPA + DHA in 12,513 patients at high cardiovascular risk but with no myocardial infarction for a median of 5 years (86). However, in a prespecified subgroup analysis, compared with placebo, EPA + DHA resulted in an 18% lower incidence of the revised primary outcome among women (composite of the time to death from cardiovascular causes or first hospital admission for cardiovascular causes). Also, admissions for heart failure were significantly lower in the long-chain omega-3 fatty acid group. It is worth noting that the trials of EPA + DHA have used a lower dose (840 mg/day) than trials of pure EPA (1.8 or 3.6 g/day), so any difference in findings of these trials could relate to dosing.

One trial that has questioned the impact of the combination of EPA + DHA is the Long Term Outcomes Study to Assess Statin Residual Risk with Epanova in High Cardiovascular Risk Patients with Hypertriglyceridemia (STRENGTH) trial (87). In this study, patients with hypertriglyceridemia and high cardiovascular risk on statin therapy were treated with 4 g/day of an oil containing EPA and DHA (as free fatty acids); this provided about 2.2 g EPA and 0.8 g DHA daily. Corn oil was used as placebo. There was no significant difference in a composite outcome of major adverse cardiovascular events among patients who received additional omega-3 fatty acids to usual background therapies vs. control, and the trial was stopped early (87). The Omega-3 Fatty Acids in Elderly with Myocardial Infarction (OMEMI) trial randomized a total of 1,027 patients with a recent myocardial infarction (in the previous 2–8 weeks) to receive approximately 1.6 g/day of EPA + DHA (930 mg EPA and 660 mg DHA as triglycerides) or corn oil (placebo) as an addition to standard care (88). After 2 years of follow-up, there was no significant difference between the two groups in the primary composite cardiovascular outcome.

Thus, REDUCE-IT and EVAPORATE both report benefits of pure EPA while STRENGTH and OMEMI report no benefit of the combination of EPA + DHA. The reasons for this discrepancy between REDUCE-IT and STRENGTH have been discussed elsewhere (89, 90) and include choice of placebo, formulation of omega-3 fatty acids (ethyl ester vs. free fatty acids) and exact omega-3 dose (3.6 vs. 3 g/day). Another possibility is that DHA negates the benefits of EPA so that the combination of EPA + DHA is less effective than EPA alone, although the mechanisms for how this would occur are unclear. There are no long-term studies comparing the therapeutic effect of pure EPA, pure DHA and the combination of EPA + DHA on hard cardiovascular endpoints.

## Conclusion

The results of this systematic review suggest that EPA and DHA have some similar and some different effects on cardiovascular risk factors. EPA and DHA both lower triglyceride levels with DHA most likely having a slightly greater effect. Furthermore, both EPA and DHA increase HDL2 cholesterol, which is cardioprotective, with the increase being greater with DHA. DHA appears to increase LDL cholesterol and LDL particle size which would render LDL less atherogenic. From the more limited study data, both EPA and DHA decreased some inflammatory markers and pro-inflammatory gene expression, with DHA having stronger effects. DHA may be more effective than EPA in decreasing heart rate and blood pressure. Both EPA and DHA alter platelet function decreasing thrombogenicity, although they have different actions on platelets. Both EPA and DHA decrease F2-isoprostanes, interpreted as a reduction in oxidative stress. They both decrease inflammatory gene expression and promote an anti-inflammatory oxylipin profile. These are all favorable effects with regard to cardiovascular risk. Reported effects of EPA and DHA on blood glucose are inconsistent.

Although the new data on the effects of EPA and DHA on blood lipids including triglycerides may create a clearer picture of those effects, the overall data around whether the two omega-3 fatty acids have differential effects on other cardiovascular risk factors is still inconsistent, but generally speaking there is a signal that DHA has a stronger impact than EPA. However, this updated systematic review is constrained by the small number of high quality RCTs that directly compare EPA to DHA and report on outcomes other than blood lipids. Therefore, there is a need for additional high-quality research to assess the independent effects of EPA and DHA on a range of cardiovascular risk factors (e.g., inflammation, blood pressure, vascular function, platelet function) in larger and more diverse study populations.

## Data Availability

The original contributions presented in the study are included in the article/Supplementary material, further inquiries can be directed to the corresponding author.
