# Supplementary material for: The differential effects of eicosapentaenoic acid and docosahexaenoic acid on cardiovascular risk factors: an updated systematic review of randomized controlled trials
Source: Front Nutr. 2024 Sep 30;11:1423228. doi: 10.3389/fnut.2024.1423228 (PMC11471719; doi:10.3389/fnut.2024.1423228)
Supplement: Supplementary file 1 [file Table_1.DOCX]

**Supplementary Table 1. Search strategies used for identifying possible articles for inclusion**

| **Embase Classic + Embase** |
| --- |
| 1. (EPA or EICOSAPENTAENOIC ACID or DHA or DOCOSAHEXAENOIC ACID).mp. [mp=title, abstract, heading word, drug trade name, original title, device manufacturer, drug manufacturer, device trade name, keyword heading word, floating subheading word, candidate term word] |
| 1. icosapentaenoic acid/ or docosahexaenoic acid/ |
| 1. docosahexaenoic acid/ |
| 1. (icosapentaenoate or timnodonate).mp. [mp=title, abstract, heading word, drug trade name, original title, device manufacturer, drug manufacturer, device trade name, keyword heading word, floating subheading word, candidate term word] |
| 1. (BLOOD LIPID* or LIPID* or TRIGLYCERIDE* or CHOLESTEROL or LDL or HDL or LIPOPROTEIN* or BLOOD PRESSURE or INFLAMM* or INTERLEUKIN-6 or IL-6 or C-REACTIVE PROTEIN or CRP or VASCULAR or HEART RATE or CARDIOVASCULAR or CARDIOMETABOLIC).mp. [mp=title, abstract, heading word, drug trade name, original title, device manufacturer, drug manufacturer, device trade name, keyword heading word, floating subheading word, candidate term word] |
| 1. lipid blood level/ or cholesterol blood level/ or lipoprotein blood level/ or phospholipid blood level/ or prostaglandin blood level/ or triacylglycerol blood level/ |
| 1. lipid/ |
| 1. triacylglycerol/ |
| 1. blood pressure/ |
| 1. 1 or 2 or 3 or 4 |
| 1. 5 or 6 or 7 or 8 or 9 |
| 1. 10 and 11 |
| 1. Limit 12 to (full text and human and yr=”2017 -Current”) |
| **PubMed** |
| 1. (EPA OR DHA OR EICOSAPENTAENOIC ACID OR DOCOSAHEXAENOIC ACID) AND (BLOOD LIPID OR LIPID OR TRIGLYCERIDE OR CHOLESTEROL OR LDL OR HDL OR LIPOPROTEIN OR BLOOD PRESSURE OR INFLAMM* OR INTERLEUKIN-6 OR IL-6 OR C-REACTIVE PROTEIN OR CRP OR VASCULAR OR HEART RATE OR CARDIOVASCULAR OR CARDIOMETABOLIC) 2. Limiters (Full text and randomized controlled trials) |
| **CINAHL Plus with Full Text** |
| 1. EPA OR DHA OR eicosapentaenoic acid OR docosahexaenoic acid |
| 1. BLOOD LIPID* or LIPID* or TRIGLYCERIDE* or CHOLESTEROL or LDL or HDL or LIPOPROTEIN* or BLOOD PRESSURE or INFLAMM* or INTERLEUKIN-6 or IL-6 or C-REACTIVE PROTEIN* or CRP or VASCULAR or HEART RATE or CARDIOVASCULAR or CARDIOMETABOLIC |
| 1. 1 and 2 Limiters - Full Text; Publication Date: 20170101-20231231; Randomized Controlled Trials |
